# Molecular Detection of Trypanosomatids in Rodents and Marsupials in the State of Amapá, Brazil

**DOI:** 10.3390/microorganisms13020242

**Published:** 2025-01-23

**Authors:** Lourdes Marina Bezerra Pessoa, Claudia Regina Silva, Kamila Gaudêncio da Silva Sales, Darlison Chagas de Souza, Lucas Lisboa Nunes Bonifácio, Rafaela Lira Nogueira de Luna, Filipe Dantas-Torres, Lúcio André Viana

**Affiliations:** 1 Programa de Pós-Graduação em Biodiversidade Tropical, Universidade Federal do Amapá, Macapá 68903-419, AP, Brazil; marinapess@gmail.com (L.M.B.P.); darlisondcs@hotmail.com (D.C.d.S.); lucviana74@gmail.com (L.A.V.); 2 Laboratório de Mamíferos, Instituto de Pesquisas Científicas e Tecnológicas do Estado do Amapá, Macapá 68903-329, AP, Brazil; crsilvaap@gmail.com; 3 Laboratório de Imunoparasitologia, Instituto Aggeu Magalhães, Fundação Oswaldo Cruz (FIOCRUZ), Recife 50740-465, PE, Brazil; kamilasalesg@gmail.com (K.G.d.S.S.); lucas_lisboa25@hotmail.com (L.L.N.B.); rafaelalira.luna@hotmail.com (R.L.N.d.L.)

**Keywords:** leishmaniasis, trypanosome, small mammals, public health

## Abstract

Trypanosomatids of the genera *Trypanosoma* and *Leishmania* are parasites of medical and veterinary importance that infect mammals, including humans and domestic and wild animals. Among mammals, rodents and marsupials play a crucial role in maintaining and spreading the zoonotic transmission cycle of these parasites. The present study aimed to detect the natural occurrence of *Trypanosoma* spp. and *Leishmania* spp. in rodents and marsupials in the state of Amapá, northern Brazil. In total, 137 samples were analyzed, of which 19 (6 marsupials and 13 rodents) were positive for trypanosomatid DNA. Partial sequences of the 18S rRNA gene of trypanosomatids were obtained from 10 out of 19 positive samples. Specifically, an undescribed *Trypanosoma* sp. was detected in *Marmosa demerarae*, *Marmosa murina*, *Zygodontomys brevicauda,* and *Neacomys paracou*. *Trypanosoma cruzi* was detected in a *Philander opossum*, whereas sequences close to *Trypanosoma wauwau* and *Trypanosoma freitasi* were obtained from *Didelphis imperfecta* and *N. paracou*, respectively. Finally, *Leishmania* (*Viannia*) sp. was detected in *Mesomys hispidus*, *Hylaeamys megacephalus*, and *Z. brevicauda*. The present study expands the knowledge about marsupials and rodents as hosts of trypanosomatids and emphasizes the need for further studies on the role of these animals as potential reservoirs of these parasites in the Amazon region.

## 1. Introduction

The family Trypanosomatidae (order Trypanosomatida) includes plant and animal parasites, which, according to their life cycle, can be classified as monoxenous or heteroxenous [1,2]. Among the genera that make up this family, *Trypanosoma* and *Leishmania* stand out for their importance in public health, as they include etiological agents of trypanosomiasis and leishmaniasis, respectively [3,4,5]. Diseases like African trypanosomiasis, Chagas disease, and leishmaniasis are classified as neglected tropical diseases and are of great importance to global health [6].

Leishmaniasis is notoriously relevant and can manifest clinically in three main forms: visceral leishmaniasis (VL), cutaneous leishmaniasis (CL), and mucocutaneous (MCL). The clinical form depends on several factors, including parasite species, host immunity, and the location of the lesions [7]. This disease affects the world’s poorest populations and is endemic in 99 countries throughout Asia, Africa, Europe, and the Americas, with over one million cases estimated to occur annually [3]. In the Americas, Brazil accounts for most leishmaniasis cases, being one of the countries with the highest number of cases of both VL and CL in the world [3,8,9]. Cutaneous leishmaniasis is the most common form of the disease in Brazil, and most cases are reported in the northern region, with 182,398 cases reported from 2001 to 2020 [10].

Chagas disease (CD) is a chronic, systemic, parasitic infection caused by the protozoan *Trypanosoma cruzi*, which is endemic in 21 countries in Latin America but has spread through human migration to countries in North America, Europe, Asia, and Oceania [11]. In Brazil, 1.9 to 4.6 million people are estimated to be infected with *T*. *cruzi* [12]. In 2020, 157 new cases of acute CD were reported in Brazil, with the north region concentrating 91.7% of the cases [12]. Most of these cases are reported in the states of Amapá and Pará.

Possible explanations for the high incidence of CD and leishmaniasis, as well as other diseases such as malaria, in northern Brazil are the close relationship between humans and the forest, where the natural cycle of these parasites takes place. In the forest environments, *Leishmania* spp. and *Trypanosoma* spp. are primarily maintained by a wide range of wildlife species, including small mammals. For instance, different species of rodents and marsupials have been found to be infected by *Leishmania* spp. and *Trypanosoma* spp. [13,14,15,16,17,18,19]. In this context, additional data about the diversity of mammalian hosts of *Leishmania* spp. and *Trypanosoma* spp. in northern Brazil would be valuable for a better understanding of the dynamics of pathogen transmission cycles in this region, which remains responsible for most cases of CL and acute CD in this country.

Studies concerning the diversity of trypanosomatids in small mammals in some northern states of Brazil are still scarce, and this is the case in the state of Amapá, where information on natural hosts of trypanosomatids is almost nonexistent [20]. In this context, the present work aimed to investigate the circulation of *Leishmania* spp. and *Trypanosoma* spp. in wild rodents and marsupials from Amapá.

## 2. Materials and Methods

### 2.1. Ethical Considerations

Our research was approved by the Ethics Committee for Animal Use (CEUA) of the Federal University of Amapá (CEUA no. 23/2022) and by the Chico Mendes Institute for Biodiversity Conservation (ICMBio) through Biodiversity Authorization and Information System (SISBio) (numbers 84253-1 and 74694-1).

### 2.2. Animals and Study Area

A total of 120 (70% ethanol-preserved spleen, liver, or pooled spleen and liver samples) used in the present study were obtained from 120 small mammals trapped in different municipalities of Amapá state (i.e., Santana, Mazagão, Calçoene, Oiapoque, Itaubal, Amapá and Porto Grande), and 17 samples (EDTA-blood, 70% ethanol-preserved spleen, liver, or pooled spleen and liver samples) from 17 animals captured in forest fragments in the municipalities of Macapá. Detailed information on the animals and samples are provided in the Supplementary Material (Table S1).

**Figure 1:**
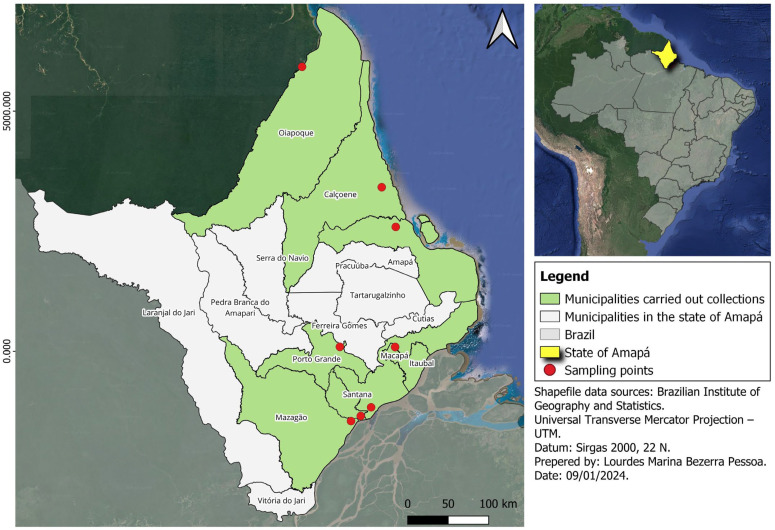
Map of the Amapá state, northern Brazil, with the municipalities of the animal trapping stations. This map was generated with QGIS v.3.28.6 (https://qgis.org, accessed on 9 January 2024).

The animals were captured by the research team of the Mastozoology Laboratory (LAMAM) of the Institute of Scientific and Technological Research of the State of Amapá (IEPA), led by the second author of this paper (C.R.S.). Captures were conducted between 2021 and 2022 in seven areas from north to south in the coastal portion of Amapá. These areas include peri-urban areas in the municipalities of Macapá (00°02′20″ N, 51°03′59″ W) and Santana (00°03′30″ S, 51°10′54″ W) and rural areas 20 km away from rural or extractive communities in different municipalities of Amapá (i.e., Santana, Mazagão, Calçoene, Oiapoque, Itaubal, Amapá, and Porto Grande) (Figure 1). The sampled areas included dryland forests, floodplain forests, savannahs, flooded environments, and riparian forests. Three complementary methodologies were used for animal trapping: live traps, pitfall traps with drift fences, and nocturnal searches.

The live traps were distributed in lines containing 12 pairs of Tomahawk and Sherman traps, placed approximately 20 m apart on the ground, and tied in the understory at a height of approximately 1.5 m, when possible. The traps were baited with peanut brittle, cornmeal, and sardines. Additionally, pitfall traps were installed with 12 60 L buckets spaced approximately 10 m apart, interconnected by a 50 cm high plastic canvas guide fence, buried approximately 5 cm deep in the ground, and held in a vertical position by fixed wooden stakes. To prevent water accumulation, the inside of the buckets had holes drilled. The sampling period varied from 8 to 12 consecutive days, and the traps were inspected daily, with bait changed in the Sherman and Tomahawk traps. The sampling effort was 3938 h for live traps (Sherman and Tomahawk) and 1008 h for pitfall traps.

The animals were transported to the Mastozoology Laboratory (LAMAM) of the Institute of Scientific and Technological Research of the State of Amapá (IEPA) within cloth bags or transport boxes. Each bag or box was labeled with location and trap type. The animals were classified by sex, size, and weight and morphologically identified. Morphological species identification was based on Patton et al. [21], Weksler and Percequillo [22], Weksler et al. [23], Voss and Jansa [24], and Voss et al. [25]. The nomenclature follows Abreu et al. [26] and Silva et al. [27].

The animals were anesthetized with hind limb intramuscular injections of ketamine hydrochloride 10% and xylazine hydrochloride 2%. However, two out of every ten specimens collected were anesthetized and euthanized with an overdose of the combination of ketamine hydrochloride 10% and xylazine hydrochloride 2% to obtain voucher specimens. These voucher specimens were deposited in the Mammal Collection of the Amapá Fauna Scientific Collection (CCFA). Fragments of liver and spleen were collected from the euthanized animals. The spleen and/or liver samples were stored in 70% ethanol for molecular analysis.

### 2.3. DNA Extraction, Nested Polymerase Chain Reaction (Nested-PCR), and Amplicon Purification

Genomic DNA was purified from blood (*n* = 8), spleen (*n* = 10), liver (*n* = 20), or pooled spleen and liver (*n* = 99) samples using commercial kits (Wizard^®^ Genomic DNA Purification Kit, Promega Corporation, Madison, WI, USA) according to manufacturer’s instructions. The quantity and purity (absorbance ratio at 260/280 nm and at 260/230 nm) of the obtained DNA were assessed using a NanoDrop 2000c Spectrophotometer (Thermo Fisher Scientific, Waltham, MA, USA), and samples were then stored at −20 °C.

A nested PCR targeting a variable region of the trypanosomatid 18S rRNA gene was carried out in two rounds. In the first round, each 25 µL reaction contained 5 μL of genomic DNA sample, 2.5 μL of DNA-free water, 12.5 μL of GoTaq^®^ Colorless Master Mix (Promega, Madison, WI, USA), and 2.5 μL of each primer at a concentration of 20 pmol/μL of the following external primers: TRY927F (5′-GAAACAAGAAACACGGGAG-3′) and TRY927R (5′-CTACTGGGCAGCTTGGA-3′) [28]. PCR products from the first amplification round were diluted (1:10) in sterile deionized water. In the second round of the nested PCR, 2 µL of this dilution was used as a template. Reactions were conducted in a final volume of 25 μL, containing 5.5 μL of DNA-free water, 12.5 μL of GoTaq^®^ Colorless Master Mix (Promega, Madison, WI, USA), and 2.5 μL of each primer at a concentration of 20 pmol/μL of the following internal primers: SSU561F (5′-TGGGATAACAAAGGAGCA-3′) and SSU561R (5′-CTGAGACTGTAACCTCAAAGC-3′) [28]. The thermocycler settings were 30 cycles at 94 °C for 30 s, 55 °C for 60 s, and 72 °C for 90 s [29]. The amplification products were stained with ethidium bromide (10 mg/mL), loaded on 1.5% agarose, and visualized by illumination with UV light.

For positive samples whose sequencing attempt with the above primers was unsuccessful, we used another nested PCR using the primers S762 (5′-GAC TTT TGC TTC CTC TAD TG-3′)/S763 (5′-CAT ATG CTT GTT TCA AGG AC-3′) targeting a ~2100 bp fragment of the 18S rRNA gene [2] in the first reaction and the primers TRnSSU-F2 (5′-GAR TCT GCG CAT GGC TCA TTA CAT CAG A-3′) and TRnSSU-R2 (5′-CRC AGT TTG ATG AGC TGC GCC T-3′) targeting a ~600 bp ([30] in the second. In the first round, each 25 µL reaction contained 5 μL of genomic DNA sample, 2.5 μL of DNA-free water, 12.5 μL of GoTaq^®^ Colorless Master Mix (Promega, Madison, MI, USA), and 2.5 μL of each primer at a concentration of 20 pmol/μL. Cycling conditions were as follows: denaturing at 94 °C for 5 min followed by 35 cycles of 94 °C for 1 min, 55 °C for 90 s, 72 °C for 90 s, and a final elongation at 72 °C for 5 min. In the second round of PCR, 1 µL of the previous reaction was used as a template. Each reaction contained a final volume of 25 μL, including 6.5 μL of DNA-free water, 12.5 μL of GoTaq^®^ Colorless Master Mix (Promega, Madison, MI, USA), and 2.5 μL of each primer at a concentration of 20 pmol/μL. Thermal cycling conditions were as follows: denaturing at 94 °C for 5 min followed by 35 cycles of 94 °C for 1 min, 64 °C for 90 s, 72 °C for 90 s, and a final elongation at 72 °C for 5 min.

A master mix without DNA (no template control, NTC) and a DNA sample extracted from cultured promastigotes of *Leishmania infantum* (positive control) were included in each assay. All reactions were run in a Veriti^®^ 96-well thermal cycler (Applied Biosystems, Foster City, CA, USA). The amplification products were stained with ethidium bromide (10 mg/mL), loaded on 1% agarose, and visualized by illumination with UV light.

### 2.4. Sequencing of Positive Samples

The amplicons were purified with the PureLink PCR Purification kit (Invitrogen, Carlsbad, CA, USA) following the manufacturer’s instructions. The purified samples were prepared using the Big Dye™ Terminator v3.1 kit (Applied Biosystems, Waltham, MA, USA) and sequenced using the ABI-PRISM 377 sequencer (Applied Biosystems, Waltham, MA, USA) in both directions (forward and reverse) with the same oligonucleotides used for the second PCR reaction. The sequences obtained were analyzed using the Geneious Prime 2025.0.2, and consensus sequences were generated considering an error probability limit of 0.05. Finally, we performed sequence similarity searches against the GenBank database using the BLASTn tool (https://blast.ncbi.nlm.nih.gov/Blast.cgi?PAGE_TYPE=BlastSearch, accessed on 11 December 2024).

### 2.5. Phylogenetic Analysis

The sequences generated herein were aligned in MEGA v11.0.13 with MUSCLE v3.8.31 [31]. For phylogenetic analyses, we also included reference 18S rRNA gene sequences from several *Trypanosoma* spp. [32]. The evolutionary history was inferred based on partial 18S gene sequences using the maximum likelihood (ML) method with IQ-TREE, with 1000 ultrafast bootstrap (UFBoot) [33,34]. The best-fit model according to the Bayesian Information Criterion (BIC) was TIM3e + I + R4. The tree was rooted against the nucleotide sequence of *Leishmania amazonensis* (GenBank accession number: JX030087). Both alignment and phylogenetic analysis were performed using MEGA v11.0.13, and the final phylogenetic tree was edited using iTOL v.6 (https://itol.embl.de, accessed on 11 December 2024).

## 3. Results

A total of 137 animals from different locations in the state of Amapá were analyzed in the present study, of which 72 (52.5%) were males, 64 (46.7%) females, and 1 undetermined (0.8%). Eighty-six animals (62.8%) were rodents, and fifty-one (37.2%) were marsupials. These animals belonged to 19 genera and 25 different species (Supplementary Material: Table S1).

Among the 137 samples analyzed, 19 (13.9%; 95% CI: 8.6–20.8%) were positive in nested PCR (Supplementary Material: Figures S1 and S2), including six marsupials and 13 rodents (Table 1). Partial 18S rRNA gene sequences were successfully obtained from ten (52.6%) (six rodents and four marsupials). Concerning the genera, *Leishmania* spp. was detected in three rodent species, and *Trypanosoma* spp. was detected in seven animals.

**Figure 2:**
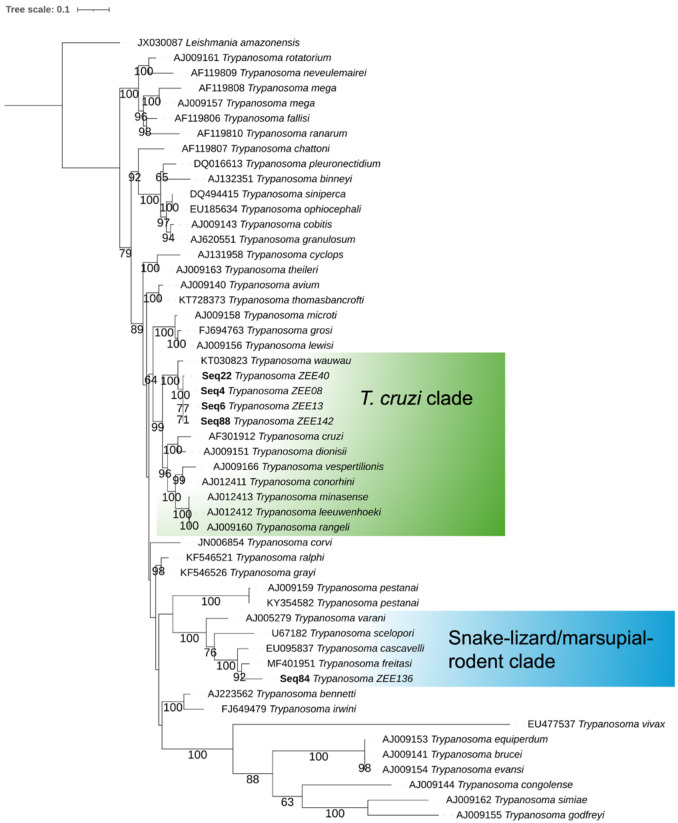
Phylogenetic reconstruction of the genus *Trypanosoma* based on partial 18S rRNA gene sequences. The dataset included 52 sequences and 2447 nucleotide sites. The tree was inferred using the maximum-likelihood method with ultrafast bootstrap (1000 replicates) and the model TIM3e + I + R4. Bootstrap values < 60 were omitted. *Leishmania amazonensis* (GenBank accession number: JX030087) was used as an outgroup. The final figure was edited with iTOL v.6. The *Trypanosoma cruzi* and the snake-lizard/marsupial-rodent clade are with different colors for better visualization.

Specifically, using the first nested PCR, an undescribed *Trypanosoma* sp. was identified in a woolly mouse opossum (*Marmosa demerarae*), a Linnaeus’s mouse opossum (*Marmosa murina*), a short-tailed cane mouse (*Zygodontomys brevicauda*), and a Paracou bristly mouse (*Neacomys paracou*). These four sequences of *Trypanosoma* sp. clustered together in the *T. cruzi* clade (Figure 2), being positioned near but separated from *Trypanosoma wauwau* with strong bootstrap support (100%) (Figure 2). The other sequence, which was obtained from a Paracou bristly mouse, clustered with *Trypanosoma freitasi* in the snake-lizard/marsupial-rodent clade (Figure 2). Finally, we obtained *Leishmania (Viannia*) sp. sequences (100% identity with JX030135, GQ332355, and JN003595) from a large-headed rice rat (*Hylaeamys megacephalus*) and a short-tailed cane mouse.

The three PCR amplification products whose sequencing failed with the first nested PCR were successfully sequenced with the second nested PCR. One sequence from a gray four-eyed opossum (*Philander opossum*) corresponded to *Trypanosoma cruzi* (100% identity with KX007998), and another from a Ferreira’s spiny tree-rat (*Mesomys hispidus*) to *a Leishmania (Viannia*) sp. (100% identity with JX030135, GQ332355, JN003595). Finally, a sequence related to *T. wauwau* (99.4% identity with KR65321) was obtained from a Guianan white-eared opossum (*Didelphis imperfecta*). All data regarding the sequence similarities are described in Table 2.

## 4. Discussion

In this study, we evaluated the occurrence of trypanosomatids in small mammals in Amapá, northern Brazil. Nineteen (13%) positive samples were detected by nested PCR, of which ten (52.6%) were successfully sequenced. These included a sequence of *T. cruzi* and three of *Leishmania (Viannia*) sp. The remaining sequences presented high percent identity and/or phylogenetic relatedness with either *T. wauwau* or *T. freitasi.*

In particular, the presence of *Leishmania (Viannia*) sp. was confirmed in three species of rodents: *Z. brevicauda*, *M. hispidus,* and *H. megacephalus*. Rodents are probably the most studied animals in terms of *Leishmania* spp. infection under both natural and experimental conditions [15]. Several species of wild rodents have been found infected by *Leishmania* spp. [35,36,37], and there is compelling experimental evidence indicating that many of them participate actively in the life cycle of these parasites in nature [38,39,40,41]. DNA sequencing attempts to identify the *Leishmania* (*Viannia*) sp. detected herein using more discriminatory targets (e.g., the heat shock protein 70 gene) were unsuccessful, probably due to the low amount of parasite DNA. Our sequences clearly belonged to the subgenus *Viannia*, which includes *L.* (*V.*) *braziliensis*, the most common species in rodents in Brazil [15]. While we cannot be sure, the species detected herein in *M. hispidus*, *H. megacephalus*, and *Z. brevicauda* could be *L.* (*V.*) *braziliensis*. Further investigations in the study area are needed to confirm this hypothesis. It is important to state that the occurrence of various *Leishmania* (*Viannia*) spp. has been recorded in different locations of Amapá, such as *L.* (*V.*) *braziliensis*, *L.* (*V.*) *guyanensis*, and *L.* (*V.*) *naiffi* responsible for CL in humans, with a high prevalence for *L.* (*V.*) *guyanensis* [42].

The rodents *Z. brevicauda* and *M. hispidus* had not yet been described as naturally infected by *Leishmania* (*Viannia*) sp. [15] and represent new host records. Interestingly, a study conducted in Colombia suggested that the role of *Z. brevicauda* as a potential reservoir for *L. infantum* should be further investigated, as this species is abundant in some areas of transmission [43]. Concerning *H. megacephalus*, a study reported a high infection rate in the central Amazon region [37], suggesting a possible role of this animal as a reservoir of *Leishmania* parasites in some areas of northern Brazil. To date, a total of 267 species of rodents are known to occur in Brazil, being associated with different habitats (semiaquatic, terrestrial, and semi-fossorial) [27,44]. The wide distribution of rodents, their abundance, and nocturnal habits may partly explain why they are frequently exposed to various *Leishmania* spp. in nature [15].

*Trypanosoma* spp. was confirmed in seven animals, including four marsupials (*D. imperfecta*, *P. opossum*, *M. demerarae*, and *M. murina*) and three rodents (two specimens of *N. paracou* and one of *Z. brevicauda*). Concerning the identity of these parasites, our analysis revealed the presence of an undescribed *Trypanosoma* sp. in marsupials (*M. demerarae* and *M. murina*) and rodents (*Z. brevicauda* and *N. paracou*). This trypanosome has previously been detected in *D. albiventris* in Mato Grosso, Brazil [45], and was phylogenetically related to *T. wauwau* (Figure 2). As a corollary, using a different nested PCR, we also obtained a longer sequence (802 bp) with 99.4% identity with *T. wauwau* (KR653210) in a Guianan white-eared opossum. *Trypanosoma wauwau* was originally described in bats in Brazil [46,47] but had not yet been recorded in non-flying mammals. The presence of *Trypanosoma* spp. in new hosts and regions is commonly reported in Brazil [48,49,50]. Incidentally, the success of adaptation of a parasite to new hosts and environments is directly related to its genotypic plasticity [51], and this is also true for the genus *Trypanosoma* [52,53,54,55]. Further studies will be necessary to ascertain whether this *Trypanosoma* sp. is, in fact, a genotype of *T. wauwau* or a close but different species. This will be pivotal for a formal delineation of the parasite detected herein.

Rodents and marsupials are recognized as important hosts in the transmission cycle of several species of trypanosomatids and play a role in maintaining these parasites in the wild [18]. Although marsupials and rodents have already been found naturally infected by several *Trypanosoma* spp. in the Neotropical region [14,15,16,17,18,19,49,56], it is common to find species that have not yet been described. This study reinforces the need for new studies on trypanosomatids of marsupials and rodents in this region.

Some species of marsupials of the family Didelphidae are considered important reservoirs of *T. cruzi* due to their ability to serve as a source of infection for the triatomine vectors [13,14,16,18,56,57,58,59,60]. This is the case of the gray four-eyed opossum (*P. opossum*), which is an important reservoir of *T. cruzi* of this parasite in some areas of the Brazilian Amazon [13,18]. Indeed, we found a gray four-eyed opossum naturally infected with *T. cruzi* in Macapá city, which reported >80% of the cases of acute Chagas disease in Amapá state from 2017 to 2022 [61]. This marsupial has terrestrial and arboreal habits and can be found in a wide variety of habitats, from more preserved areas to degraded areas [13,62]. This probably favors its exposure to several species of parasites, including *T*. *cruzi*.

## 5. Conclusions

This study reports the occurrence of trypanosomatids in different species of rodents and marsupials in the eastern Brazilian Amazon, revealing new host–parasite associations and expanding our understanding of the host diversity of *Leishmania* spp. and *Trypanosoma* spp. in small mammals in Brazil. Our results may contribute to future studies on the transmission pattern of trypanosomatids, especially those of zoonotic concern. Further research to characterize the *Leishmania (Viannia*) sp. found herein and to investigate the role of *Z. brevicauda*, *M. hispidus* and *H. megacephalus* in the transmission cycle of this species is needed.

## Figures and Tables

**Table 1:** Small mammals (rodents and marsupials) tested molecularly for trypanosomatids using nested PCR.

Order	Species	Total Tested	Number of Positive (Type of Sample)	Positivity (%)
Didelphimorphia	*Cryptonanus* sp.	6	0	0.0
	*Didelphis imperfecta*	1	1 (pooled spleen and liver)	100.0
	*Didelphis marsupialis*	9	0	0.0
	*Gracilinanus emiliae*	2	0	0.0
	*Hyladelphys kalynowskii*	1	0	0.0
	*Marmosa demerarae*	3	1 (liver)	33.3
	*Marmosa murina*	15	2 (both liver)	13.3
	*Metachirus nudicaudatus*	1	1 (pooled spleen and liver)	100.0
	*Monodelphis touan*	8	0	0.0
	*Philander opossum*	5	1 (pooled spleen and liver)	20.0
Rodentia	*Dactylomys dactylinus*	2	0	0.0
	*Hylaeamys megacephalus*	19	2 (both pooled spleen and liver)	10.5
	*Mesomys hispidus*	3	1 (pooled spleen and liver)	33.3
	*Neacomys paracou*	8	4 (1 liver, 3 pooled spleen and liver)	50.0
	*Oecomys auyantepui*	1	0	0.0
	*Oecomys bicolor*	2	1 (pooled spleen and liver)	50.0
	*Oecomys rutilus*	2	0	0.0
	*Oecomys* sp.	1	0	0.0
	*Proechimys cuvieri*	15	1 (pooled spleen and liver)	6.7
	*Proechimys guyannensis*	20	1 (pooled spleen and liver)	5.0
	*Rattus rattus*	1	0	0.0
	*Rhypidomys nitela*	1	0	0.0
	*Sigmodon alstoni*	2	0	0.0
	*Zygodontomys brevicauda*	9	3 (1 spleen, 2 pooled spleen and liver)	33.3
Total		137	19 (4 liver, 1 spleen, 14 pooled spleen and liver)	13.9

**Table 2:** Small mammals (rodents and marsupials) molecularly positive for trypanosomatids using PCR and BLASTn results.

Species	Common Name	Municipality	Sample ID	Sample	Sequence Size (Our Genbank Accession Number) and BLASTn Results, with Highest Percent Identity and Species (GenBank Accession Number)
*Didelphis imperfecta*	Guianan white-eared opossum	Macapá	RAM 07 (21)	Spleen and liver (pooled)	802 bp (PQ766626), 99.4% identity with *Trypanosoma wauwau* (KR653210)
*Hylaeamys megacephalus*	Large-headed rice rat	Oiapoque	ZEE 132 (83)	Spleen and liver (pooled)	523 bp (PQ766617), 100% identity with *Leishmania (Viannia*) spp. (JX030135, GQ332355, JN003595)
*Marmosa demerarae*	Woolly mouse opossum	Mazagão	ZEE 08 (4)	Liver	540 bp (PQ766614), 99.4% identity with *Trypanosoma* sp. (MN196493)
*Marmosa murina*	Linnaeus’s mouse opossum	Mazagão	ZEE 13 (6)	Liver	532 bp (PQ766615), 99.8% identity with *Trypanosoma* sp. (MN196493)
*Mesomys hispidus*	Ferreira’s spiny tree-rat	Macapá	RAM 11 (26)	Spleen and liver (pooled)	894 bp (PQ766627), 99.9% identity with different *Leishmania (Viannia*) spp. (JX030135, GQ332355, JN003595)
*Neacomys paracou*	Paracou bristly mouse	Oiapoque	ZEE 136 (84)	Spleen and liver (pooled)	450 bp (PQ799498), 99.1% identity with *Trypanosoma freitasi* (MF401951)
*Neacomys paracou*	Paracou bristly mouse	Oiapoque	ZEE 142 (88)	Spleen and liver (pooled)	534 bp (PQ766619), 99.8% identity with *Trypanosoma* sp. (MN196493)
*Philander opossum*	Gray four-eyed opossum	Macapá	RAM 15 (30)	Spleen and liver (pooled)	882 bp (PQ766628), 100% identity with *Trypanosoma cruzi* (KX007998)
*Zygodontomys brevicauda*	Short-tailed cane mouse	Mazagão	ZEE 16B (8)	Spleen	525 bp (PQ766616), 99.6% identity with *Leishmania (Viannia*) spp. (JX030135, GQ332355, JN003595)
*Zygodontomys brevicauda*	Short-tailed cane mouse	Calçoene	ZEE 40 (22)	Spleen and liver (pooled)	532 bp (PQ766618), 99.6% identity with *Trypanosoma* sp. (MN196493)

## Data Availability

Data supporting the conclusions of this study are included in the manuscript and associated files. Sequences generated herein are available in GenBank (accession numbers: PQ766614-PQ766619, PQ766626-PQ766628).

## Supplementary Materials

The following supporting information can be downloaded at: https://www.mdpi.com/article/10.3390/microorganisms13020242/s1, Table S1. Mammals captured in the Amapá state and included in this study. Figure S1. Representative gel with some of the small mammal samples positive ate the first nested-PCR. Figure S2. Gel with the three small mammal samples that were positive in the second nested-PCR assay.

## References

[1] Kaufer A., Ellis J., Stark D., Barratt J. (2017). The evolution of trypanosomatid taxonomy. Parasit. Vectors..

[2] Maslov D.A., Lukeš J., Jirků M., Simpson L. (1996). Phylogeny of trypanosomes as inferred from the small and large subunit rRNAs: Implications for the evolution of parasitism in the trypanosomatid protozoa. Mol. Biochem. Parasitol..

[3] Alvar J., Vélez I.D., Bern C., Herrero M., Desjeux P., Cano J., Jannin J., Boer M.D., WHO Leishmaniasis Control Team (2012). Leishmaniasis worldwide and global estimates of its incidence. PLoS ONE.

[4] Cucunubá Z.M., Gutiérrez-Romero S.A., Ramírez J.D., Velásquez-Ortiz N., Ceccarelli S., Parra-Henao G., Henao-Martínez A.F., Rabinovich J., Basáñez M.G., Nouvellet P. (2024). The epidemiology of Chagas disease in the Americas. Lancet Reg. Health Am..

[5] Lindner A.K., Lejon V., Barrett M.P., Blumberg L., Bukachi S.A., Chancey R.J., Edielu A., Matemba L., Mesha T., Mwanakasale V. (2024). New WHO guidelines for treating rhodesiense human African trypanosomiasis: Expanded indications for fexinidazole and pentamidine. Lancet Infect. Dis..

[6] WHO (2024). Neglected Tropical Diseases. https://www.who.int/health-topics/neglected-tropical-diseases#tab=tab_1.

[7] WHO Leishmaniasis. 2023. Health-Topics/Leishmaniasis. https://www.who.int/news-room/fact-sheets/detail/leishmaniasis.

[8] Maia-Elkhoury A.N., Yadón Z.E., Díaz M.I.S., Lucena F.F.A.L., Castellanos L.G., Sanchez-Vazquez M.J. (2016). Exploring spatial and temporal distribution of cutaneous Leishmaniasis in Americas, 2001–2011. PloS Negl. Trop. Dis..

[9] Portella T.P., Kraenkel R.A. (2021). Spatial-temporal pattern of cutaneous leishmaniasis in Brazil. Infect. Dis. Poverty..

[10] Belo V.S., Bruhn F.R.P., Barbosa D.S., Câmara D.C.P., Simões T.C., Buzanovsky L.P., Duarte A.G.S., de Melo S.N., Cardoso D.T., Donato L.E. (2023). Temporal patterns, spatial risks, and characteristics of tegumentary leishmaniasis in Brazil in the first twenty years of the 21st Century. PLoS Negl. Trop. Dis..

[11] De Sousa A.S., Vermeij D., Ramos A.N.J.R., Luquetti A.O. (2024). Chagas disease. Lancet.

[12] BRASIL (2022). Casos de Doença de Chagas Aguda (DCA) Segundo Unidade Federada de Infecção e ano de Início de Sintomas, Brasil, 2010 a 2020. https://www.gov.br/saude/pt-br/assuntos/saude-de-a-a-z/d/doenca-de-chagas/arquivos/casos-de-doenca-de-chagas-aguda-dca-segundo-unidade-federada-de-infeccao-e-ano-de-inicio-de-sintomas-brasil-2010-a-2020.pdf.

[13] Roque A.L.R., Xavier S.C.C., Gerhardt M., Silva M.F.O., Lima V.S., D’Aandrea P.S., Jansen A.M. (2013). *Trypanosoma cruzi* among wild and domestic mammals in different areas of the Abaetetuba municipality (Pará State, Brazil), an endemic Chagas disease transmission area. Vet. Parasitol..

[14] Cassia-Pires R., Boite M.C., D’Andrea P.S., Herrera H.M., Cupolillo E., Jansen A.M., Roque L.R. (2014). Distinct *Leishmania* Species Infecting Wild Caviomorph Rodents (Rodentia: Hystricognathi) from Brazil. PLoS Negl. Trop. Dis..

[15] Roque A., Jansen A. (2014). Wild and synanthropic reservoirs of *Leishmania* species in the Americas. J. Parasitol. Parasites Wildl..

[16] Caldart E.T., Freire R.L., Ferreira F.P., Ruffolo B.B., Sbeghen M.R., Mareze M., Garcia J.L., Mitsuka-Breganó R., Navarro I.T. (2017). *Leishmania* in synanthropic rodents (*Rattus rattus*): New evidence for the urbanization of *Leishmania (Leishmania) amazonensis*. Braz. J. Vet. Parasitol..

[17] Morales E.A., Mayor P., Bowler M., Aysanoa E., Pérez-Velez E.S., Pérez J., Ventocilla J.A., Baldeviano C.C., Lescano A.G. (2017). Prevalence of *Trypanosoma cruzi* and Other Trypanosomatids in Frequently-Hunted Wild Mammals from the Peruvian Amazon. Am. J. Trop. Med. Hyg..

[18] Jansen A.M., Xavier S.C.C., Roque A.L.R. (2018). *Trypanosoma cruzi* transmission in the wild and its most important reservoir hosts in *Brazil*. Parasit. Vectors..

[19] Berbigier A.P., Barros J.H.D.S., Pontes E.S., Lisboa C.V., Gentile R., Xavier S.C.D.C., Jansen A.M., Roque A.L.R. (2021). Trypanosomatid Richness in Wild and Synanthropic Small Mammals from a Biological Station in Rio de Janeiro, Brazil. Pathogens.

[20] Moreno E., Sabioni L., Seixas M., Souza Filho J., Marcelino A., Shimabukuro P. (2019). Evidence of a sylvatic enzootic cycle of *Leishmania infantum* in the State of Amapá, Brazil. Rev. Soc. Bras. Med. Trop..

[21] Patton J.L., Pardiñas U.F., D’Elía G. (2015). Mammals of South America. v. 2. Rodents.

[22] Weksler M., Percequillo A.R. (2011). Key to the genera of the Tribe Oryzomyini (Rodentia: Cricetidae: Sigmodontinae). Mastozool. neotrop..

[23] Weksler M., Percequillo A.R., Voss R.S. (2006). Ten New Genera of Oryzomyine Rodents (Cricetidae: Sigmodontinae). Am. Mus. Novit..

[24] Voss R.S., Jansa S.A. (2009). Phylogenetic relationships and classification of *Didelphis marsupials*, an extant radiation of new world metatherian mammals. Bull. Am. Mus. Nat. Hist..

[25] Voss R.S., Lunde D.P., Simmons N.B. (2001). The mammals of Paracou, French Guiana: A Neotropical lowland rainforest fauna. Part-2. Nonvolant species. Bull. Am. Mus. Nat. Hist..

[26] Abreu E.F., Casali D., Costa-Araújo R., Garbino G.S.T., Libardi G.S., Loretto D., Loss A.C., Marmontel M., Moras L.M., Nascimento M.C. (2022). Lista de Mamíferos do Brasil (2022-1).

[27] Silva C.R., Martins A.M., Castro I.J., Bernard E., Cardoso E.M., Lima D.S., Gregorin R., Rossi R.V., Percequillo A.R., Castro K.C. (2013). Mammals of Amapá State, eastern Brazilian Amazonia: A revised taxonomic list with comments on species distributions. Mammalia.

[28] Noyes H.A., Stevens J.R., Teixeira M., Phelan J., Holz P. (1999). A nested PCR for the ssrRNA gene detects *Trypanosoma binneyi* in the platypus and *Trypanosoma* sp. in wombats and kangaroos in Australia. Int. J. Parasitol..

[29] Smith A., Clark P., Averis S., Lymbery A.J., Wayne A.F., Morris K.D., Thompson R.C. (2008). Trypanosomes in a declining species of threatened Australian marsupial, the brush-tailed bettong *Bettongia penicillate* (Marsupialia: Potoroidae). Parasitology.

[30] Seward E.A., Votýpka J., Kment P., Lukeš J., Kelly S. (2017). Description of *Phytomonas oxycareni* n. sp. from the salivary glands of *Oxycarenus* lavaterae. Protist.

[31] Edgar R.C. (2004). MUSCLE: Multiple sequence alignment with high accuracy and high throughput. Nucleic Acids Res..

[32] Borges A.R., Engstler M., Wolf M. (2021). 18S rRNA gene sequence-structure phylogeny of the Trypanosomatida (Kinetoplastea, Euglenozoa) with special reference to *Trypanosoma*. Eur. J. Protistol..

[33] Hoang D.T., Chernomor O., Von Haeseler A., Minh B.Q., Vinh L.S. (2018). UFBoot2: Improving the ultrafast bootstrap approximation. Mol. Biol. Evol..

[34] Minh B.Q., Nguyen M.A., Von Haeseler A. (2013). Ultrafast approximation for phylogenetic bootstrap. Mol. Biol. Evol..

[35] Brandão-Filho S.P., Brito M.E., Carvalho F.G., Ishikawa E.A., Cupolillo E., Floeter-Winter L., Shaw J.J. (2003). Wild and synanthropic hosts of *Leishmania (Viannia) braziliensis* in the endemic cutaneous leishmaniasis locality of Amaraji, Pernambuco State, Brazil. Trans. R. Soc. Trop. Med. Hyg..

[36] Lima B.S., Dantas-Torres F., de Carvalho M.R., Marinho-Junior J.F., de Almeida E.L., Brito M.E., Gomes F., Brandão-Filho S.P. (2013). Small mammals as hosts of *Leishmania* spp. in a highly endemic area for zoonotic leishmaniasis in North-Eastern Brazil. Trans. R. Soc. Trop. Med. Hyg..

[37] Achilles G.R., Kautzmann R.P., Chagas H.D.F., Pereira-Silva J.W., Almeida J.F., Fonseca F.R., Da Silva M.N.F., Pessoa F.A.C., Nava A.F.D., Ríos-Velásquez C.M. (2021). Presence of trypanosomatids, with emphasis on *Leishmania*, in Rodentia and Didelphimorphia mammals of a rural settlement in the central Amazon region. Mem. Inst. Oswaldo Cruz.

[38] Andrade M.S., Courtenay O., Brito M.E.F., Carvalho F.G., Carvalho A.W.S., Soares F., Carvalho S.M., Costa P.L., Zampieri R., Floeter-Winter L.M. (2015). Infectiousness of Sylvatic and Synanthropic Small Rodents Implicates a Multi-host Reservoir of *Leishmania (Viannia) braziliensis*. PLoS Negl. Trop. Dis..

[39] Shaw J.J., Marinho-Júnior J.F., Courtenay O., Brandão-Filho S.P. (2023). Assessing reservoir host status in leishmaniasis with special reference to the infectiousness of *Leishmania (Viannia) braziliensis* infections in wild rodents. Rev. Soc. Bras. Med. Trop..

[40] Marinho-Júnior J.F., Monteiro J.F.C.L.S., Sales De Carvalho A.W., De Carvalho F.G., De Paiva Cavalcanti M., Shaw J., Courtenay O., Brandão-Filho S.P. (2023). High levels of infectiousness of asymptomatic *Leishmania (Viannia) braziliensis* infections in wild rodents highlights their importance in the epidemiology of American Tegumentary Leishmaniasis in Brazil. PLoS. Negl. Trop. Dis..

[41] Courtenay O., Marinho-Júnior J.F., Brito M.E.F., Monteiro J.F.C.L.S., Shaw J.J., Brandão-Filho S.P. (2023). Incidence of Human and Free-Ranging Wild Rodent Infections with *Leishmania (Viannia) braziliensis*, Aetiological Agent of Cutaneous Leishmaniasis. Pathogens.

[42] Almeida A.N.F., Nascimento L.C.S.D., Sousa E.S.M.M., Oliveira A.J.D., Sena M.G., Resende B.M., Chaves R.C.G., Garcez L.M. (2020). Surveillance of cutaneous leishmaniasis in clinical samples: Distribution of *Leishmania guyanensis* in the state of Amapá, Brazil, 2018. Epidemiol. Serv. Saude..

[43] López M., Erazo D., Hoyos J., Léon C., Fuya P., Lugo L., Cordovez J.M., González C. (2021). Measuring spatial co-occurrences of species potentially involved in *Leishmania* transmission cycles through a predictive and fieldwork approach. Sci. Rep..

[44] Bonvicino C.R., Oliveira J.A., D’andrea P.S. (2008). Guia dos Roedores do BRASIL, Com Chaves Para Gêneros Baseadas em Caracteres Externos.

[45] Nantes W.A.G., Santos F.M., De Macedo G.C., Barreto W.T.G., Gonçalves L.R., Rodrigues M.S., Chulli J.V.M., Rucco A.C., Assis W.O., Porfírio G.E.O. (2021). Trypanosomatid species in *Didelphis albiventris* from urban forest fragments. Parasitol. Res..

[46] Lima L., Espinosa-Alvarez O., Pinto C.M., Cavazzana M., Pavan A.C., Carranza J.C., Lim B.K., Campaner M., Takata C.S.A., Camargo E.P. (2015). New insights into the evolution of the *Trypanosoma cruzi* clade provided by a new trypanosome species tightly linked to Neotropical *Pteronotus* bats and related to na Australian lineage of trypanosomes. Parasit. Vectors.

[47] Da Costa A.P., Nunes P.H., Leite B.H.S., Ferreira J.I.G.D.S., Tonhosolo R., Da Rosa A.R., Rocha P.A., Aires C.C., Gennari S.M., Marcili A. (2016). Diversity of bats trypanosomes in hydroeletric area of Belo Monte in Brazilian Amazonia. Acta Trop..

[48] Rangel D.A., Lisboa C.V., Novaes R.L.M., Silva B.A., Souza R.D.F., Jansen A.M., Moratelli R., Roque A.L.R. (2019). Isolation and characterization of trypanosomatids, including *Crithidia mellificae*, in bats from the Atlantic Forest of Rio de Janeiro, Brazil. PLoS Negl. Trop. Dis..

[49] Rodrigues M.S., Lima L., Xavier S.C.C., Herrera H.M., Rocha F.L., Roque A.L.R., Teixeira M.M.G., Jansen A.M. (2019). Uncovering *Trypanosoma* spp. diversity of wild mammals by the use of DNA from blood clots. Parasites Wildl..

[50] Santos F.M., Barreto W.T.G., Macedo G.C., Barros J.H.S., Xavier S.C.C., Garcia C.M., Mourão G., Oliveira J., Rimoldi A.R., Porfírio G.E.O. (2019). The reservoir system for *Trypanosoma* (Kinetoplastida, Trypanosomatidae) species in large Neotropical wetland. Acta Trop..

[51] Agosta S.J. (2006). On ecological fitting, plant–insect associations, herbivore host shifts, and host plant selection. Oikos.

[52] Câmara A.C., Varela-Freire A.A., Valadares H.M., Macedo A.M., D’Avila D.A., Machado C.R., Lages-Silva E., Chiari E., Galvão L.M. (2010). Genetic analyses of *Trypanosoma cruzi* isolates from naturally infected triatomines and humans in northeastern Brazil. Acta Trop..

[53] Cordon-Obras C., Cano J., González-Pacanowska D., Benito A., Navarro M., Bart J.M. (2013). *Trypanosoma brucei gambiense* adaptation to different mammalian sera is associated with VSG expression site plasticity. PLoS ONE.

[54] Drini S., Criscuolo A., Lecha T.P., Imamura H., Skalický T., Rachidi N., Lukeš J., Dujardin J.C., Späth G.F. (2016). Species- and strainspecific adaptation of the HSP70 super family in pathogenic Trypanosomatids. Genome Biol. Evol..

[55] Jaimes-Dueñez J., Cantillo-Barraza O., Triana-Chávez O., Mejia-Jaramillo A.M. (2020). Molecular surveillance reveals bats from eastern Colombia infected with *Trypanosoma theileri* and *Trypanosoma wauwau*-like parasites. Prev. Vet. Med..

[56] Lopes C.M.T., Barreto R.F.S.M., Pavan M.G., Pereira C.S., Roque A.L.R. (2018). *Trypanosoma janseni* n. sp. (Trypanosomatida: Trypanosomatidae) isolated from *Didelphis aurita* (Mammalia: Didelphidae) in the Atlantic Rainforest of Rio de Janeiro, Brazil: Integrative taxonomy and phylogeography within the *Trypanosoma cruzi* clade. Memórias Inst. Oswaldo Cruz.

[57] Legey A.P., Pinho A.P., Xavier S.C.C., Leon L., Jansen A.M. (1999). Humoral immune response kinetics in *Philander opossum* and *Didelphis marsupialis* infected and immunized by *Trypanosoma cruzi*. Mem. Inst. Oswaldo Cruz.

[58] Barros J.H.S., Xavier S.C.C., Bilac D., Lima V.S., Dario M.A., Jansen A.M. (2017). Identification of novel mammalian hosts and Brazilian biome geographic distribution of *Trypanosoma cruzi* TcIII and TcIV. Acta Trop..

[59] Bezerra-Santos M.A., Ramos R.A.N., Campos A.K., Dantas-Torres F., Otranto D. (2021). *Didelphis* spp. opossums and their parasites in the Americas: A One Health perspective. Parasitol. Res..

[60] Martinez Ibarra J.A., Martinez B.O., Rodas Martinez A.Z., Flores R.A., Garcia C.I.M., Franco E.R., Villalobos G., Martinez Hernandez F. (2024). *Trypanosoma cruzi* in Wild and Synanthropic Mammals in Two Regions of Mexico: A Fieldwork and Genetic Discrete Typing Unit Review. Vector Borne Zoonotic Dis..

[61] Carvalho G.H.F., Medeiros G.G., Magalhães R.L.B. (2024). Subnotificação de doença de Chagas no Estado do Amapá no período da pandemia de COVID-19. Cad. Pedagógico.

[62] Ardente N.C., Ferreguetti A.C., Gettinger D., Leal P., Mendes-Oliveira A.C., Martins-Hatano F., Bergallo H.G. (2016). Diversity and impacts of mining on the nonvolant small mammal communities of two vegetation types in the Brazilian Amazon. PLoS ONE.

