# Supplementary material for: Molecular Detection of Trypanosomatids in Rodents and Marsupials in the State of Amapá, Brazil
Source: Microorganisms. 2025 Jan 23;13(2):242. doi: 10.3390/microorganisms13020242 (PMC11857483; doi:10.3390/microorganisms13020242)
Supplement: Supplementary file 1 [file microorganisms-13-00242-s001.zip › microorganisms-3394824-supplementary.pdf]

Table S1. Mammals captured in the Amapá state and included in this study. The samples used and the trypanosomatid 18S rRNA PCR results are shown.

Order	Animal	Sex	Sample	PCR Results	Municipality	Coordinates_Municipality
**Didelphimorphia**	*Monodelphis touan*	M	Liver	Negative	Santana	00º 03' 30" S 51º 10' 54" W
	Monodelphis touan	M	Spleen	Negative	Santana	00º 03' 30" S 51º 10' 54" W
	Cryptonanus sp.	F	Spleen and liver (pooled)	Negative	Mazagão	00º 06' 54" S 51º 17' 22" W
	Marmosa demerarae	F	Liver	Positive	Mazagão	00º 06' 54" S 51º 17' 22" W
	Marmosa murina	M	Liver	Negative	Mazagão	00º 06' 54" S 51º 17' 22" W
	Marmosa murina	M	Liver	Positive	Mazagão	00º 06' 54" S 51º 17' 22" W
	Monodelphis touan	M	Liver	Negative	Mazagão	00º 06' 54" S 51º 17' 22" W
	Marmosa murina	M	Liver	Positive	Mazagão	00º 06' 54" S 51º 17' 22" W
	Marmosa murina	M	Liver	Negative	Mazagão	00º 06' 54" S 51º 17' 22" W
	Metachirus nudicaudatus	M	Spleen and liver (pooled)	Positive	Calçoene	02º 29' 51" N 50º 56' 55" W
	Philander opossum	F	Spleen and liver (pooled)	Negative	Calçoene	02º 29' 51" N 50º 56' 55" W
	Marmosa murina	F	Spleen and liver (pooled)	Negative	Calçoene	02º 29' 51" N 50º 56' 55" W
	Didelphis marsupialis	F	Spleen and liver (pooled)	Negative	Calçoene	02º 29' 51" N 50º 56' 55" W
	Marmosa murina	F	Spleen and liver (pooled)	Negative	Calçoene	02º 29' 51" N 50º 56' 55" W
	Philander opossum	M	Spleen and liver (pooled)	Negative	Calçoene	02º 29' 51" N 50º 56' 55" W
	Marmosa murina	M	Liver	Negative	Itaubal	00º 42' 42" N 50º 48' 00" W
	Marmosa murina	M	Spleen	Negative	Itaubal	00º 42' 42" N 50º 48' 00" W
	Didelphis marsupialis	F	Spleen and liver (pooled)	Negative	Itaubal	00º 42' 42" N 50º 48' 00" W
	Monodelphis touan	M	Spleen and liver (pooled)	Negative	Itaubal	00º 42' 42" N 50º 48' 00" W
	Marmosa demerarae	F	Spleen and liver (pooled)	Negative	Amapá	02º 03' 12" N 50º 47' 35" W
	Monodelphis touan	F	Spleen	Negative	Oiapoque	03º 50' 35" N 51º 50' 06" W
	Monodelphis touan	N/D	Spleen and liver (pooled)	Negative	Oiapoque	03º 50' 35" N 51º 50' 06" W
	Philander opossum	F	Spleen and liver (pooled)	Negative	Oiapoque	03º 50' 35" N 51º 50' 06" W
	Philander opossum	M	Spleen and liver (pooled)	Negative	Oiapoque	03º 50' 35" N 51º 50' 06" W

	Marmosa demerarae	F	Spleen and liver (pooled)	Negative	Oiapoque	03º 50' 35" N 51º 50' 06" W
	Didelphis marsupialis	F	Spleen and liver (pooled)	Negative	Oiapoque	03º 50' 35" N 51º 50' 06" W
	Monodelphis touan	F	Spleen and liver (pooled)	Negative	Porto Grande	00º 42' 48" N 51º 24' 48" W
	Cryptonanus sp.	F	Spleen and liver (pooled)	Negative	Porto Grande	00º 42' 48" N 51º 24' 48" W
	Hyladelphys kalynowskii	M	Spleen and liver (pooled)	Negative	Porto Grande	00º 42' 48" N 51º 24' 48" W
	Monodelphis touan	F	Spleen and liver (pooled)	Negative	Porto Grande	00º 42' 48" N 51º 24' 48" W
	Gracilinanus emiliae	M	Spleen and liver (pooled)	Negative	Porto Grande	00º 42' 48" N 51º 24' 48" W
	Cryptonanus sp.	M	Spleen and liver (pooled)	Negative	Porto Grande	00º 42' 48" N 51º 24' 48" W
	Cryptonanus sp.	M	Spleen and liver (pooled)	Negative	Porto Grande	00º 42' 48" N 51º 24' 48" W
	Marmosa murina	M	Spleen and liver (pooled)	Negative	Porto Grande	00º 42' 48" N 51º 24' 48" W
	Cryptonanus sp.	M	Spleen and liver (pooled)	Negative	Porto Grande	00º 42' 48" N 51º 24' 48" W
	Gracilinanus emiliae	M	Spleen and liver (pooled)	Negative	Porto Grande	00º 42' 48" N 51º 24' 48" W
	Cryptonanus sp.	F	Spleen and liver (pooled)	Negative	Porto Grande	00º 42' 48" N 51º 24' 48" W
	Didelphis marsupialis	F	Blood	Negative	Macapá	00º 02' 20" N 51º 03' 59" W
	Didelphis marsupialis	M	Blood	Negative	Macapá	00º 02' 20" N 51º 03' 59" W
	Marmosa murina	M	Spleen and liver (pooled)	Negative	Macapá	00º 02' 20" N 51º 03' 59" W
	Marmosa murina	M	Spleen and liver (pooled)	Negative	Macapá	00º 02' 20" N 51º 03' 59" W
	Didelphis imperfecta	M	Spleen and liver (pooled)	Positive	Macapá	00º 02' 20" N 51º 03' 59" W
	Marmosa murina	F	Blood	Negative	Macapá	00º 02' 20" N 51º 03' 59" W
	Marmosa murina	M	Blood	Negative	Macapá	00º 02' 20" N 51º 03' 59" W
	Marmosa murina	F	Spleen and liver (pooled)	Negative	Macapá	00º 02' 20" N 51º 03' 59" W
	Didelphis marsupialis	M	Spleen and liver (pooled)	Negative	Macapá	00º 02' 20" N 51º 03' 59" W
	Philander opossum	M	Spleen and liver (pooled)	Positive	Macapá	00º 02' 20" N 51º 03' 59" W
	Didelphis marsupialis	F	Blood	Negative	Macapá	00º 02' 20" N 51º 03' 59" W
	Didelphis marsupialis	F	Blood	Negative	Macapá	00º 02' 20" N 51º 03' 59" W
	Marmosa murina	M	Blood	Negative	Macapá	00º 02' 20" N 51º 03' 59" W
	Didelphis marsupialis	M	Blood	Negative	Macapá	00º 02' 20" N 51º 03' 59" W

**Rodentia**	*Hylaeamys megacephalus*	F	Liver	Negative	Mazagão	00º 06' 54" S 51º 17' 22" W
	Zygodontomys brevicauda	M	Spleen	Positive	Mazagão	00º 06' 54" S 51º 17' 22" W
	Zygodontomys brevicauda	M	Liver	Negative	Mazagão	00º 06' 54" S 51º 17' 22" W
	Proechimys guyannensis	F	Liver	Negative	Mazagão	00º 06' 54" S 51º 17' 22" W
	Zygodontomys brevicauda	F	Spleen and liver (pooled)	Negative	Calçoene	02º 29' 51" N 50º 56' 55" W
	Proechimys cuvieri	F	Spleen and liver (pooled)	Negative	Calçoene	02º 29' 51" N 50º 56' 55" W
	Zygodontomys brevicauda	M	Spleen and liver (pooled)	Negative	Calçoene	02º 29' 51" N 50º 56' 55" W
	Proechimys cuvieri	F	Spleen and liver (pooled)	Negative	Calçoene	02º 29' 51" N 50º 56' 55" W
	Proechimys cuvieri	F	Spleen and liver (pooled)	Negative	Calçoene	02º 29' 51" N 50º 56' 55" W
	Zygodontomys brevicauda	F	Spleen and liver (pooled)	Negative	Calçoene	02º 29' 51" N 50º 56' 55" W
	Oecomys rutilus	M	Spleen and liver (pooled)	Negative	Calçoene	02º 29' 51" N 50º 56' 55" W
	Zygodontomys brevicauda	M	Spleen and liver (pooled)	Positive	Calçoene	02º 29' 51" N 50º 56' 55" W
	Hylaeamys megacephalus	M	Spleen and liver (pooled)	Negative	Calçoene	02º 29' 51" N 50º 56' 55" W
	Proechimys cuvieri	F	Spleen and liver (pooled)	Negative	Calçoene	02º 29' 51" N 50º 56' 55" W
	Proechimys cuvieri	F	Spleen	Negative	Itaubal	00º 42' 42" N 50º 48' 00" W
	Proechimys cuvieri	F	Liver	Negative	Itaubal	00º 42' 42" N 50º 48' 00" W
	Proechimys cuvieri	M	Spleen and liver (pooled)	Negative	Itaubal	00º 42' 42" N 50º 48' 00" W
	Proechimys guyannensis	F	Spleen and liver (pooled)	Negative	Itaubal	00º 42' 42" N 50º 48' 00" W
	Hylaeamys megacephalus	F	Spleen and liver (pooled)	Positive	Itaubal	00º 42' 42" N 50º 48' 00" W
	Hylaeamys megacephalus	F	Spleen and liver (pooled)	Negative	Itaubal	00º 42' 42" N 50º 48' 00" W
	Hylaeamys megacephalus	F	Spleen and liver (pooled)	Negative	Itaubal	00º 42' 42" N 50º 48' 00" W
	Hylaeamys megacephalus	F	Spleen and liver (pooled)	Negative	Itaubal	00º 42' 42" N 50º 48' 00" W
	Hylaeamys megacephalus	M	Spleen and liver (pooled)	Negative	Itaubal	00º 42' 42" N 50º 48' 00" W
	Oecomys auyantepui	M	Liver	Negative	Itaubal	00º 42' 42" N 50º 48' 00" W
	Oecomys sp.	M	Spleen and liver (pooled)	Negative	Itaubal	00º 42' 42" N 50º 48' 00" W
	Hylaeamys megacephalus	M	Spleen and liver (pooled)	Negative	Itaubal	00º 42' 42" N 50º 48' 00" W
	Dactylomys dactylinus	M	Spleen and liver (pooled)	Negative	Itaubal	00º 42' 42" N 50º 48' 00" W

	Dactylomys dactylinus	F	Spleen and liver (pooled)	Negative	Itaubal	00º 42' 42'' N 50º 48' 00'' W
	Hylaeamys megacephalus	M	Spleen and liver (pooled)	Negative	Itaubal	00º 42' 42'' N 50º 48' 00'' W
	Hylaeamys megacephalus	F	Spleen and liver (pooled)	Negative	Itaubal	00º 42' 42'' N 50º 48' 00'' W
	Hylaeamys megacephalus	F	Spleen and liver (pooled)	Negative	Itaubal	00º 42' 42'' N 50º 48' 00'' W
	Proechimys guyannensis	F	Spleen	Negative	Itaubal	00º 42' 42'' N 50º 48' 00'' W
	Proechimys guyannensis	F	Liver	Negative	Itaubal	00º 42' 42'' N 50º 48' 00'' W
	Hylaeamys megacephalus	F	Liver	Negative	Itaubal	00º 42' 42'' N 50º 48' 00'' W
	Proechimys guyannensis	F	Spleen and liver (pooled)	Negative	Itaubal	00º 42' 42'' N 50º 48' 00'' W
	Proechimys cuvieri	F	Spleen and liver (pooled)	Negative	Itaubal	00º 42' 42'' N 50º 48' 00'' W
	Mesomys hispidus	M	Spleen and liver (pooled)	Negative	Itaubal	00º 42' 42'' N 50º 48' 00'' W
	Proechimys cuvieri	F	Spleen and liver (pooled)	Negative	Itaubal	00º 42' 42'' N 50º 48' 00'' W
	Proechimys cuvieri	M	Liver	Negative	Itaubal	00º 42' 42'' N 50º 48' 00'' W
	Proechimys cuvieri	M	Spleen	Negative	Itaubal	00º 42' 42'' N 50º 48' 00'' W
	Proechimys cuvieri	F	Spleen and liver (pooled)	Positive	Itaubal	00º 42' 42'' N 50º 48' 00'' W
	Proechimys guyannensis	M	Spleen and liver (pooled)	Negative	Itaubal	00º 42' 42'' N 50º 48' 00'' W
	Zygodontomys brevicauda	F	Spleen and liver (pooled)	Positive	Amapá	02º 03' 12'' N 50º 47' 35'' W
	Hylaeamys megacephalus	M	Spleen and liver (pooled)	Negative	Amapá	02º 03' 12'' N 50º 47' 35'' W
	Hylaeamys megacephalus	M	Spleen and liver (pooled)	Negative	Amapá	02º 03' 12'' N 50º 47' 35'' W
	Proechimys guyannensis	M	Spleen and liver (pooled)	Negative	Amapá	02º 03' 12'' N 50º 47' 35'' W
	Neacomys paracou	M	Spleen and liver (pooled)	Positive	Amapá	02º 03' 12'' N 50º 47' 35'' W
	Hylaeamys megacephalus	F	Spleen and liver (pooled)	Negative	Amapá	02º 03' 12'' N 50º 47' 35'' W
	Sigmodon alstoni	M	Spleen and liver (pooled)	Negative	Amapá	02º 03' 12'' N 50º 47' 35'' W
	Proechimys cuvieri	F	Spleen and liver (pooled)	Negative	Amapá	02º 03' 12'' N 50º 47' 35'' W
	Zygodontomys brevicauda	M	Spleen and liver (pooled)	Negative	Amapá	02º 03' 12'' N 50º 47' 35'' W
	Proechimys guyannensis	F	Spleen and liver (pooled)	Negative	Amapá	02º 03' 12'' N 50º 47' 35'' W
	Oecomys bicolor	F	Spleen and liver (pooled)	Positive	Amapá	02º 03' 12'' N 50º 47' 35'' W
	Proechimys guyannensis	F	Spleen and liver (pooled)	Negative	Amapá	02º 03' 12'' N 50º 47' 35'' W

	Hylaeamys megacephalus	F	Spleen and liver (pooled)	Negative	Amapá	02º 03' 12" N 50º 47' 35" W
	Hylaeamys megacephalus	F	Spleen and liver (pooled)	Negative	Amapá	02º 03' 12" N 50º 47' 35" W
	Proechimys guyannensis	F	Spleen and liver (pooled)	Negative	Amapá	02º 03' 12" N 50º 47' 35" W
	Proechimys guyannensis	F	Spleen and liver (pooled)	Positive	Amapá	02º 03' 12" N 50º 47' 35" W
	Proechimys guyannensis	M	Spleen and liver (pooled)	Negative	Amapá	02º 03' 12" N 50º 47' 35" W
	Sigmodon alstoni	F	Spleen and liver (pooled)	Negative	Amapá	02º 03' 12" N 50º 47' 35" W
	Oecomys bicolor	M	Spleen and liver (pooled)	Negative	Amapá	02º 03' 12" N 50º 47' 35" W
	Hylaeamys megacephalus	F	Spleen and liver (pooled)	Positive	Oiapoque	03º 50' 35" N 51º 50' 06" W
	Neacomys paracou	M	Spleen and liver (pooled)	Positive	Oiapoque	03º 50' 35" N 51º 50' 06" W
	Neacomys paracou	M	Spleen and liver (pooled)	Positive	Oiapoque	03º 50' 35" N 51º 50' 06" W
	Neacomys paracou	M	Spleen and liver (pooled)	Negative	Oiapoque	03º 50' 35" N 51º 50' 06" W
	Neacomys paracou	M	Liver	Positive	Oiapoque	03º 50' 35" N 51º 50' 06" W
	Proechimys guyannensis	F	Spleen	Negative	Oiapoque	03º 50' 35" N 51º 50' 06" W
	Neacomys paracou	M	Spleen and liver (pooled)	Negative	Oiapoque	03º 50' 35" N 51º 50' 06" W
	Hylaeamys megacephalus	M	Spleen	Negative	Oiapoque	03º 50' 35" N 51º 50' 06" W
	Proechimys cuvieri	M	Liver	Negative	Oiapoque	03º 50' 35" N 51º 50' 06" W
	Proechimys cuvieri	M	Spleen	Negative	Oiapoque	03º 50' 35" N 51º 50' 06" W
	Rhypidomys nitela	M	Spleen and liver (pooled)	Negative	Porto Grande	00º 42' 48" N 51º 24' 48" W
	Proechimys guyannensis	M	Spleen and liver (pooled)	Negative	Porto Grande	00º 42' 48" N 51º 24' 48" W
	Proechimys guyannensis	M	Spleen and liver (pooled)	Negative	Porto Grande	00º 42' 48" N 51º 24' 48" W
	Proechimys guyannensis	F	Spleen and liver (pooled)	Negative	Porto Grande	00º 42' 48" N 51º 24' 48" W
	Zygodontomys brevicauda	M	Spleen and liver (pooled)	Negative	Porto Grande	00º 42' 48" N 51º 24' 48" W
	Neacomys paracou	F	Spleen and liver (pooled)	Negative	Porto Grande	00º 42' 48" N 51º 24' 48" W
	Neacomys paracou	M	Liver	Negative	Porto Grande	00º 42' 48" N 51º 24' 48" W
	Oecomys rutilus	M	Liver	Negative	Porto Grande	00º 42' 48" N 51º 24' 48" W
	Proechimys guyannensis	M	Spleen and liver (pooled)	Negative	Porto Grande	00º 42' 48" N 51º 24' 48" W
	Proechimys guyannensis	F	Spleen and liver (pooled)	Negative	Porto Grande	00º 42' 48" N 51º 24' 48" W

	Rattus rattus	F	Spleen and liver (pooled)	Negative	Porto Grande	00º 42' 48" N 51º 24' 48" W
	Proechimys guyannensis	M	Spleen and liver (pooled)	Negative	Macapá	00º 02' 20" N 51º 03' 59" W
	Mesomys hispidus	F	Spleen and liver (pooled)	Positive	Macapá	00º 02' 20" N 51º 03' 59" W
	Mesomys hispidus	M	Spleen and liver (pooled)	Negative	Macapá	00º 02' 20" N 51º 03' 59" W
	Nectomys rattus	M	Spleen and liver (pooled)	Negative	Macapá	00º 02' 20" N 51º 03' 59" W

legends: M: male, F: female, N/D: not determined

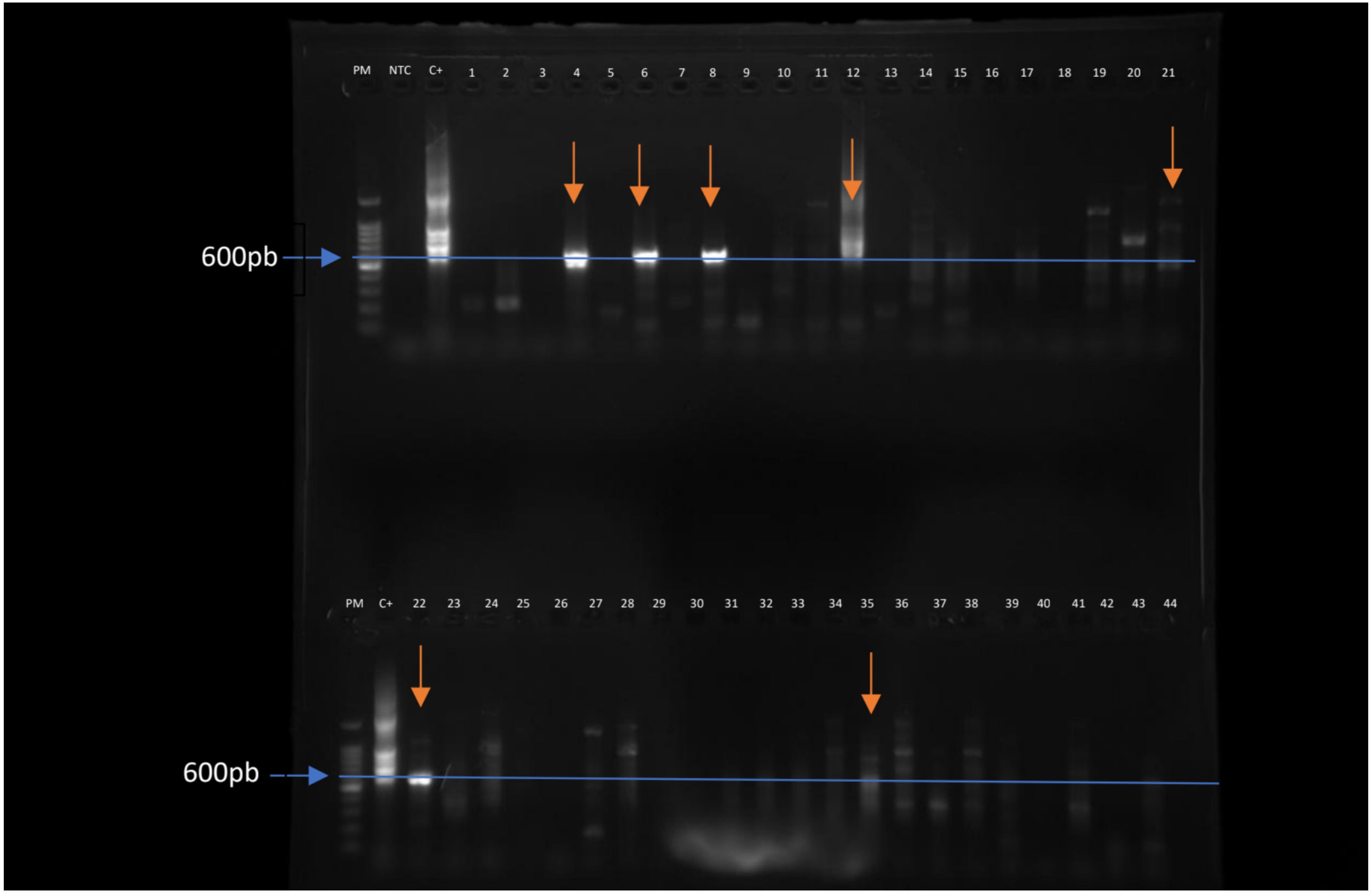

Figure S1. Representative gel with some of the small mammal samples positive ate the first nested-PCR.
Note: For more details, see Methods, Table 2, Figure 1 and Table S1.

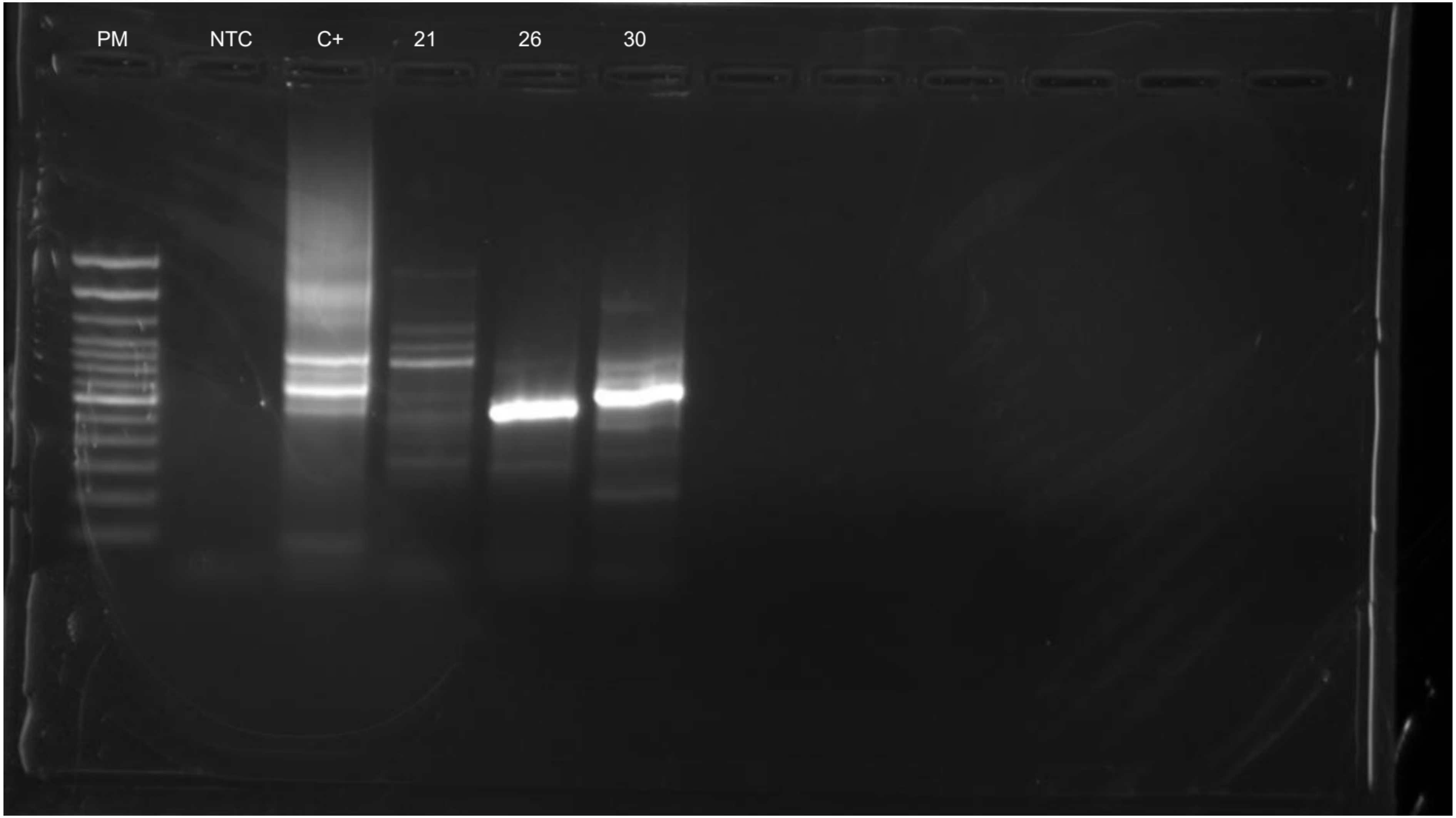

Figure S2. Gel with the three small mammal samples that were positive in the second nested-PCR assay.
Note: For more details, see Methods, Table 2, Figure 1 and Table S1.
